# QUAD shot: an effective cyclical hypofractionated palliative radiotherapy for salivary gland carcinoma

**DOI:** 10.1259/bjrcr.20190132

**Published:** 2020-06-12

**Authors:** Ryo Toya, Tetsuo Saito, Tomohiko Matsuyama, Takahiro Watakabe, Kohsei Yamaguchi, Daizo Murakami, Yumi Honda, Sachiko Mizutari, Yorihisa Orita, Natsuo Oya

**Affiliations:** 1 Department of Radiation Oncology, Faculty of Life Sciences, Kumamoto University, Kumamoto, Japan; 2 Department of Otolaryngology-Head and Neck Surgery, Faculty of Life Sciences, Kumamoto University, Kumamoto, Japan; 3 Department of Diagnostic Pathology, Kumamoto University Hospital, Kumamoto, Japan; 4 Department of Otolaryngology, Kumamoto Red Cross Hospital, Kumamoto, Japan

## Abstract

Surgery with or without post-operative radiotherapy is the mainstay treatment for salivary gland carcinoma (SGC); however, palliative radiotherapy or supportive observation is considered for elderly patients. An 87-year-old female who was diagnosed with SGC in the left parotid gland, with a clinical stage T4aN2bM0 Stage IVA, underwent the Radiation Therapy Oncology Group 8502 “QUAD shot” regimen (14.8 Gy/4 fractions, twice-daily treatment with a 6 h interval, on 2 consecutive days), which were repeated every 4 weeks 3 times using volumetric modulated arc therapy. During and after the treatment, she experienced no acute toxicity but had Grade 1 xerostomia. At 4 months after completion of the treatment, [^18^F]-fluoro-2-deoxy-D-glucose positron emission tomography/CT revealed a complete metabolic response to the treatment. She is still alive without any evidence of recurrence 9 months after completion of the treatment. The Radiation Therapy Oncology Group 8502 “QUAD shot” regimen using VMAT may be an effective palliative treatment for SGC with minimal toxicity.

## Introduction

Salivary gland carcinomas (SGCs) are relatively rare, accounting for approximately 5% of all head and neck cancers (HNC).^1^ The general management of SGC in most patients includes surgery followed by radiotherapy (RT) for unfavorable prognostic factors such as T3 or T4 tumors, close or incomplete resection margins, high grade, perineural invasion, and positive lymph nodes.^2^ Primary RT is considered for technically inoperable cases and patients at high risk of complications because of comorbidities or advanced age, or when the patient refuses surgery. The total dose of 66–70 Gy with elective nodal irradiation in 6–7 weeks is recommended for definitive RT; however, acute adverse effects of mucositis and dermatitis significantly decrease the quality of life of the patients.^3^ Therefore, patients with refusal or inability to tolerate the length of treatment and toxicities from RT are not eligible for definitive RT. We report a case of SGC that achieved a complete metabolic response (CMR) on [^18^F]-fluoro-2-deoxy-D-glucose (FDG) positron emission tomography (PET)/CT images at 4 months after the completion of the cyclical hypofractionated palliative RT known as the Radiation Therapy Oncology Group (RTOG) 8502 “QUAD shot” regimen using volumetric modulated arc therapy (VMAT).

## Clinical presentation

**Figure 1.**
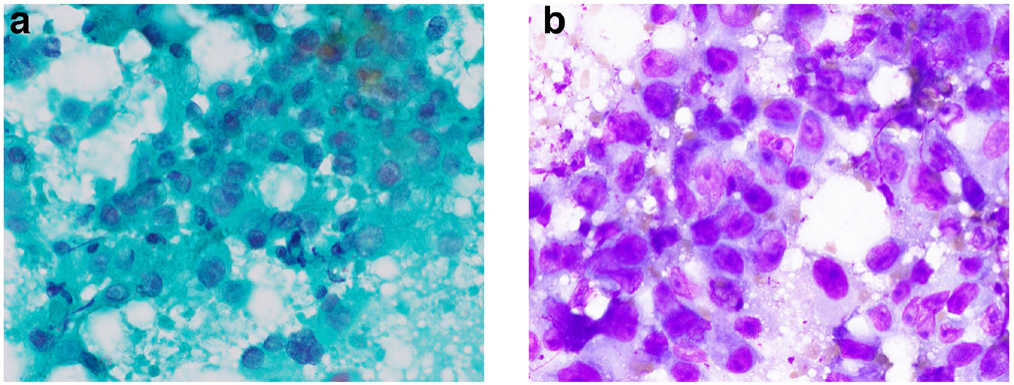
Pretreatment cytology with Papanicolaou (a) and Giemsa stain (b).

**Figure 2.**
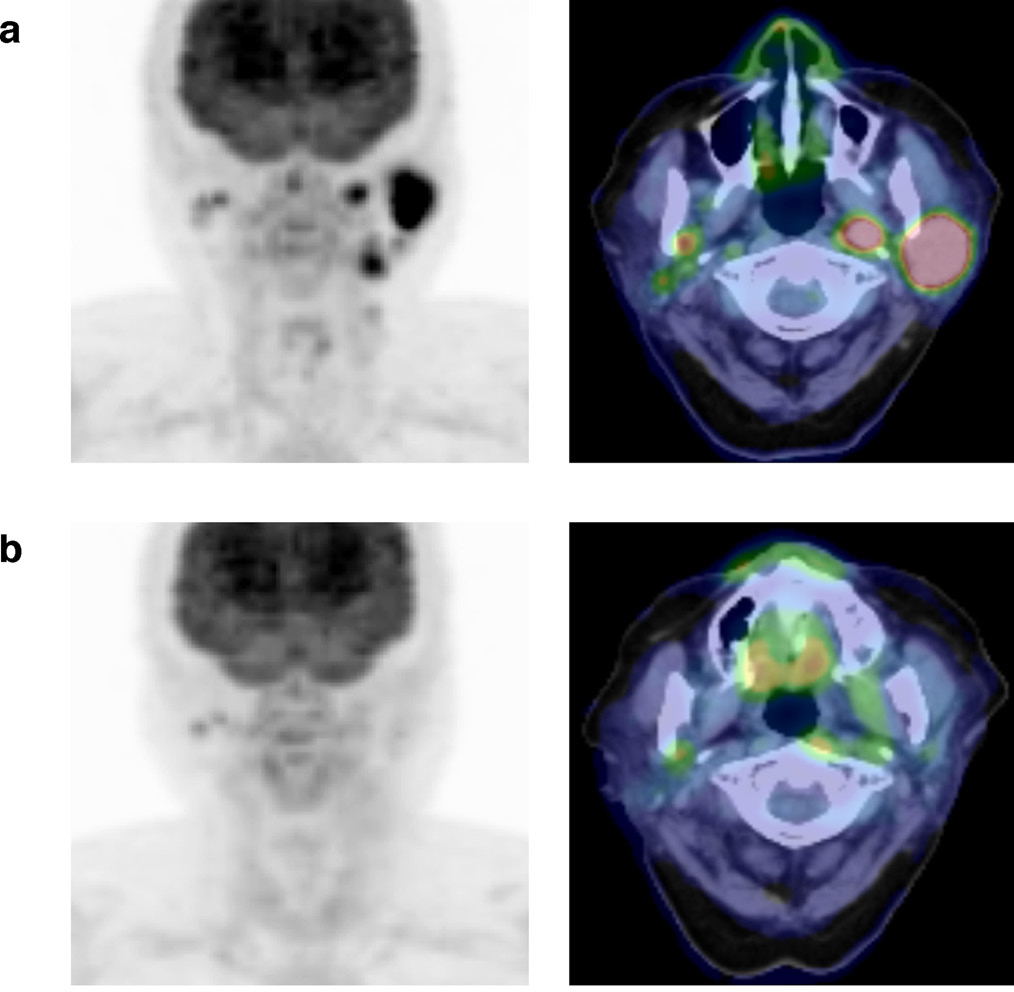
Images of pre-treatment ^18^F-FDG PET/CT (a), and images of post-treatment ^18^F-FDG PET/CT obtained at 4 months after the completion of the RTOG 8502 regimen (b). ^18^F-FDG PET, [^18^F]-fluoro-2-deoxy-D-glucose positron emission tomography

An 87-year-old previously healthy female was referred to Kumamoto University Hospital for treatment of a tumor in the left parotid gland. She presented with facial pain due to the tumor. Analysis of the fine-needle aspiration biopsy specimen confirmed it was a carcinoma and was suspicious for a salivary duct carcinoma (Figure 1). Contrast-enhanced magnetic resonance and ^18^F-FDG PET/CT images suggested the facial nerve invasion of the primary tumor and multiple lymph node metastases in the left neck node levels II and VII (Figure 2). From these findings, the tumor was diagnosed as cT4aN2bM0 Stage IVA SGC according to the eighth edition of the Union for International Cancer Control TNM classification. We offered treatment options for palliative RT with the RTOG 8502 regimen in addition to supportive observation because she refused to undergo surgery and definitive RT owing to advanced age. After obtaining fully informed consent, we performed RT with the RTOG 8502 regimen according to her wishes.

## Treatment

**Figure 3.**
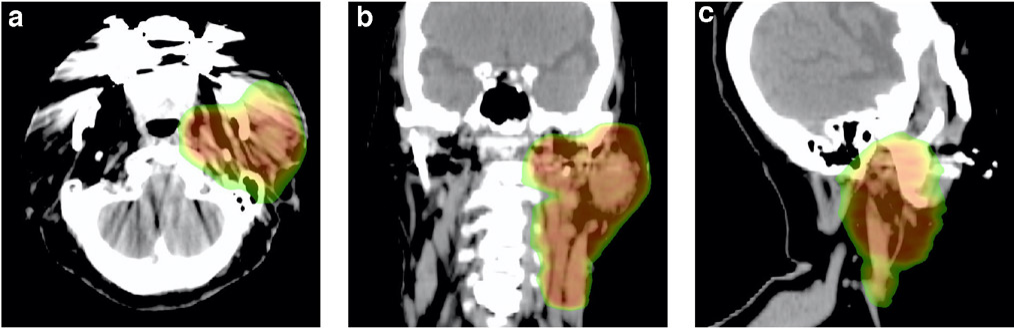
Dose distribution of volumetric modulated arc therapy in axial (a), coronal (b), and sagittal views (c).

Before each RT cycle, the patient was simulated with CT imaging in a dedicated thermoplastic head and neck mask for immobilization. Primary tumor and the involved lymph nodes were included in the gross tumor volume (GTV). A clinical target volume margin of 5 mm was added to the GTV for subclinical invasion, whereas planning target volume margins of 3 mm were added to cover setup errors. RT plans were designed to use VMAT (RapidArc; Varian Medical Systems, Palo Alto, CA) with 6 MV photons generated by a linear accelerator (Clinac iX; Varian Medical Systems, Palo Alto, CA) with one arc rotating from 181° to 179° in a clockwise direction and the dose rate varying between 0 and 600 MU/min. RT was administered using the RTOG 8502 “QUAD shot” regimen (14.8 Gy/4 fractions, twice-daily treatment with a 6 h interval, on two consecutive days), which were repeated every 4 weeks for three cycles (Figure 3).^4–7^ She did not receive systemic therapy.

She completed all three cycles of the treatment with total RT dose of 44.4 Gy. During and after treatment, she experienced a noticeable decrease in pain and no acute toxicity but did have Grade 1 xerostomia. At 4 months after completion of the treatment, she underwent ^18^F-FDG PET/CT imaging. The complete disappearance of ^18^F-FDG accumulation was observed and the treatment response was considered as CMR (Figure 2). She is currently alive with no evidence of progression 9 months after completion of the treatment.

## Discussion

Palliative RT or supportive observation is considered for patients who are unfit for definitive RT, especially for elderly patients. Severe RT toxicities should be avoided when treatment is for palliation.^3^ Although a once-daily hypofractionated RT plan with a 30 Gy dose in 10 fractions is commonly performed as a palliative RT for various tumor sites, this treatment plan is unsuitable for HNC owing to its acute adverse effects. Chen et al.^8^ reported that the frequency of acute toxicities of Grade 3 and higher was more than 40% with this treatment regimen for patients with HNC.

The RTOG performed a Phase II study of RT in the 1980s that included 2 days of twice-daily fractionation with a fraction size of 3.7 Gy (14.8 Gy per cycle) repeated at 3–6 week intervals for a total of three cycles with an RT dose of 44.4 Gy for pelvic malignancies.^4^ Thereafter, this RTOG 8502 “QUAD shot” regimen was introduced into head and neck malignancies. Paris et al.^7^ treated 37 patients with advanced head and neck malignancies with the RTOG 8502 regimen in a Phase I-II study. The radiation was delivered using a two-dimensional (2D) RT technique and 21 (57%) patients completed all three cycles. Of the 39 lesions, 30 (77%) achieved a tumor response (complete or partial response). Acute toxicities were acceptable and no late complications were observed. The average survival after completion of RT was 4.5 months. Corry et al.^9^conducted a Phase II study wherein they treated 30 patients with advanced head and neck squamous cell carcinoma using a similar QUAD shot regimen with a 3.5 Gy dose per fraction, differing slightly from the original RTOG 8502 regimen. The RT delivery was performed using a 2D-RT technique. A total of 16 (53%) patients completed all three cycles in this study; 16 (53%) patients achieved a tumor response. Of the 27 evaluable patients, Grade 2 mucositis and salivary gland toxicity were observed in 3 (11%) and 10 (37%), respectively. No patient experienced Grade 3 or higher adverse events. The median overall (OS) and progression-free survival (PFS) were 5.7 and 3.1 months, respectively.

In the last two decades, the technical development of RT techniques, such as three-dimensional conformal RT (3D-CRT) and intensity-modulated radiotherapy (IMRT), has provided an enhanced dose concentration to the target volumes with reduced dose to organs at risk (OARs).^10,11^ In the 2000s, these RT techniques were introduced into palliative RT for HNC. Lok et al.^5^ performed RT using the RTOG 8502 regimen for 75 patients with HNC, including 7 (9%) patients with SGC. IMRT was utilized in 55 (73%) patients and 41 (55%) patients underwent IMRT in the first cycle. 28 (36%) patients completed at least three cycles and 22 (29 %) patients underwent concurrent chemotherapy with at least one cycle. They reported that the tumor response or relief of the presenting symptoms was observed in 49 (65%) patients. The rate of Grades 2 and 3 acute toxicities were observed in 28 and 5%, respectively. The median OS was 5.67 months. Gamez et al.^6^ performed RT with the RTOG 8502 regimen with carboplatin or cetuximab for 21 patients with advanced HNC. 3D-CRT and IMRT were performed in 6 (29%) and 15 (71%) patients, respectively. Sixteen (76%) patients completed all three cycles. They reported that tumor responses and relief of the presenting symptoms were observed in 18 of 21 patients (86%) and in all of 16 (100%) patients, respectively. They observed Grade 2 mucositis or xerostomia in 7 (35%) patients and no Grade 3 or higher acute toxicities. Median OS and PFS were 7 and 4 months, respectively. These reports suggested that the use of 3D-CRT and/or IMRT for the treatment delivery of the RTOG 8502 regimen may provide appropriate palliative effects with minimum toxicities, although concurrent systemic therapy was performed. The RTOG 8502 regimen delivered by 3D-CRT or IMRT is recommended as a palliative RT regimens for HNC by the current National Comprehensive Cancer Network guidelines.^3^

More recently, VMAT has been introduced for the treatment of HNC.^12^ In VMAT, multileaf collimator positions, dose rate, and gantry rotation speed are allowed to vary during treatment. In comparison with conventional fixed-field IMRT, VMAT provides similar dose distribution to the target volume with a reduced dose to OARs. Moreover, the time of the treatment session for VMAT is much shorter than that for conventional fixed-field IMRT; the approximate treatment times are 2–4 and 10–15 min for VMAT and conventional fixed-field IMRT, respectively.^12,13^ The candidates for palliative RT often have advanced age, poor performance status, and symptoms such as pain, bleeding, and dysphagia. Therefore, a prolonged treatment time may lead to some distress.^5,6,13,14^ Introduction of VMAT into performing the RTOG 8502 regimen may be a reasonable palliative treatment for HNC in terms of not only a dosimetric benefit but also avoiding discomfort during treatment delivery.

SGC is known to be a radioresistance tumor,^15^ but our case suggested that the RTOG 8502 regimen using VMAT may be effective for SGC with minimum toxicities. This regimen is worth considering as palliative therapy instead of supportive observation for not only squamous cell carcinoma but also other types of carcinoma.

## Learning points

1. Here, we report a case of SGC that achieved a complete metabolic response on ^18^F-FDG PET/CT images with minimal toxicities after the completion of the RTOG 8502 “QUAD shot” regimen.
2. This regimen using VMAT is worth considering as palliative therapy instead of supportive observation for SGC.
